# Efficacy of a topical combination of esafoxolaner, eprinomectin and praziquantel against *Amblyomma maculatum* infestations in cats[Fn FN1]

**DOI:** 10.1051/parasite/2024044

**Published:** 2024-08-07

**Authors:** Eric Tielemans, Carin Rautenbach, Zamantungwa Khumalo, Frederic Beugnet

**Affiliations:** 1 Boehringer Ingelheim Animal Health 29 avenue Tony Garnier 69007 Lyon France; 2 Clinvet International (Pty) Ltd. P.O. Box 11186 Universitas, Bloemfontein 9321 Republic of South Africa

**Keywords:** *Amblyomma maculatum*, Cat, Efficacy, Esafoxolaner

## Abstract

*Amblyomma maculatum*, the Gulf Coast tick, infests a wide range of vertebrate species including livestock, dogs, cats, and humans. It is a species of significant veterinary and public health importance, especially as a vector of diseases, for instance American canine hepatozoonosis or tidewater spotted fever. An experimental study was conducted to evaluate the efficacy of NexGard^®^ Combo, a topical endectoparasiticide product for cats combining eprinomectin, praziquantel and esafoxolaner, against induced infestations of *A. maculatum* in cats. This Good Clinical Practice (GCP) study used a randomized, negative controlled, masked design. Ten cats were allocated to an untreated group and ten to a treated group, dosed once on Day 0 at the minimum label dose. On Days −2, 7, 14, 21, 28, 35, and 42, cats were infested with ~50 unfed adult *A. maculatum.* On Days 3, 10, 17, 24, 31, 38, and 45, i.e., 72 h after treatment and subsequent infestations, ticks were removed, counted and the numbers of live attached tick in each group were used for efficacy calculations. At each time-point, all untreated cats were adequately infested, demonstrating a vigorous tick population and an adequate study model. The curative efficacy after a single application against existing tick infestation, 72 h after treatment, was 98.7%. The preventive efficacy, 72 h after weekly infestations, over the following five weeks ranged from 93.8% to 99.4%.

## Abstract (fr)

*Amblyomma maculatum*, la tique de la Gulf Coast, infeste un large éventail d’espèces de vertébrés, notamment le bétail, les chiens, les chats et les humains. Il s’agit d’une espèce d’importance significative en médecine vétérinaire et en santé publique, notamment en tant que vecteur de maladies, par exemple l’hépatozoonose canine américaine ou la fièvre pourprée des marées. Une étude expérimentale a été menée pour évaluer l’efficacité de NexGard^®^ Combo, un produit endectoparasiticide topique pour chats associant éprinomectine, praziquantel et esafoxolaner, contre les infestations par *A. maculatum* provoquées chez le chat. Cette étude de bonnes pratiques cliniques (BPC) a utilisé une conception randomisée, contrôlée négativement et masquée. Dix chats ont été répartis dans un groupe non traité et dix chats dans un groupe traité, traités une fois au jour 0 à la dose minimale indiquée sur l’étiquette. Aux jours −2, 7, 14, 21, 28, 35 et 42, les chats ont été infestés par environ 50 *A. maculatum* adultes non nourris. Les jours 3, 10, 17, 24, 31, 38 et 45, c’est-à-dire 72 heures après le traitement et les infestations ultérieures, les tiques ont été retirées, comptées et le nombre de tiques vivantes attachées dans chaque groupe a été utilisé pour les calculs d’efficacité. À chaque instant, tous les chats non traités étaient correctement infestés, démontrant une population de tiques vigoureuse et un modèle d’étude adéquat. L’efficacité curative après une seule application contre une infestation de tiques existante, 72 heures après le traitement, était de 98,7%. L’efficacité préventive, 72 heures après les infestations hebdomadaires, au cours des cinq semaines suivantes, variait entre 93,8% et 99,4%.

## Introduction

*Amblyomma maculatum* (Koch, 1844), the ‘Gulf Coast Tick’, is a three-host Ixodidae infesting a wide range of vertebrate species. Immature forms mainly feed on birds and small mammals, while adults feed on larger mammals including livestock, dogs, cats, and wildlife (e.g., coyotes, skunks, panthers, and bears). Humans may occasionally be infested. The Gulf Coast tick is well adapted to coastal lands, woodlands, and xerophilic environments [8, 20, 29, 34]. It was historically described in coastal regions of Texas, South Carolina, and parts of Central and South America, and mainly in livestock [12, 20, 29]. Over the last few decades an expansion to the Northeast and Midwest USA, for example in Kansas and Oklahoma, has been observed [13, 17, 19, 28], likely through livestock population movement, wildlife population interactions, namely birds, coyotes, deer, and through climatic change [1–3, 30, 31, 33]. It is a tick species of significant veterinary and public health importance. The bites of *A. maculatum* can cause marked and painful inflammatory reactions with consequent edema, abscesses, and predisposition for myiasis and secondary infections [10, 23, 29]. Gulf Coast ticks preferentially infest the ears of host animals. In ruminants, infestations of the external ear commonly cause ear deformation, swelling, and drooping described as ‘gotch ear’ [10]; severe infestations may cause lethargy and weakness [34]. Importantly, *A. maculatum* is a vector of several human and animal pathogens [14–16, 32]. In humans, *A. maculatum* is described as the main vector of *Rickettsia parkeri*, the agent of ‘Tidewater spotted fever’, an eschar-associated febrile illness [9, 20, 22–24]. In animals, *A. maculatum* is the main vector of *Hepatozoon americanum* the agent of the American canine hepatozoonosis [5, 11, 25]. Feline hepatozoonosis has been reported in regions where the canine disease is present, but the feline *Hepatozoon* species have not been formally identified and their vector is not described [4]. Other pathogens of importance transmitted by *A. maculatum* are *Rickettsia felis* (feline rickettsiosis), *Leptospira pomona* (livestock leptospirosis), and *Ehrlichia ruminantium* (heartwater in livestock) [32].

NexGard^®^ Combo is a topical endectoparasiticide product for cats combining esafoxolaner, an isoxazoline compound with insecticidal and acaricidal activity, eprinomectin and praziquantel, nematicidal and cestodicidal compounds, respectively. This product was designed to provide a broad spectrum parasiticide solution for cats, as mixed infestations are common in this species [6, 7, 21]. This product was already demonstrated to be efficacious against *Ixodes ricinus, Ixodes scapularis,* the temperate lineage of *Rhipicephalus sanguineus*, and *Amblyomma americanum* [26–28, 35].

This manuscript describes a study conducted to evaluate the efficacy of NexGard^®^ Combo against induced infestations with *A. maculatum* in cats.

## Materials and methods

### Ethics

Animals were managed with due regard for their wellbeing and the study designs were reviewed and approved by the Sponsor and local Institutional Animal Care and Use Committees.

### Study design

This study, conducted in 2023, was designed in accordance with Good Clinical Practices as described in International Cooperation on Harmonization of Technical Requirements for Registration of Veterinary Medicinal Products (VICH) guideline GL9 and was designed in accordance with the World Association for the Advancement of Veterinary Parasitology (W.A.A.V.P.) guidelines for evaluating the efficacy of parasiticides for the treatment, prevention, and control of flea and tick infestation on dogs and cats [18].

This study was conducted under a randomized design, based on a pre-treatment live attached *A. maculatum* count, 48 h after infestation. The efficacy assessment was based on comparison of live attached ticks found in an untreated control group and a treated group at identical weekly time-points after treatment. All personnel collecting animal health and efficacy data were masked to treatment assignment.

### Animals and husbandry

Twenty purpose-bred, healthy domestic shorthair cats, ten males and ten females, aged 1.5–5.5 years old and weighing 3.1–5.3 kg were included in the study. To avoid inter-animal treatment contamination, cats were single housed, with visual and auditory contact with conspecifics. Cats were housed in an indoor, environmentally controlled unit. Cats were fed a daily ration of age-appropriate certified commercial cat diet (Hill’s Science Plan adult cat food with chicken) and had permanent access to potable water.

### *Amblyomma maculatum* isolate

The *A. maculatum* ticks had originally been collected by Arachni-Med Research LLC (Sunbury, Ohio, USA) in 2021 from the field in Hutchinson, Reno County, Kansas, USA. The colony was obtained by the test facility in March 2022 where it was maintained on rabbits not previously treated with acaricides. At least two generations were produced at the test facility before use.

### Treatment

Cats were treated once on Day 0. NexGard^®^ Combo was applied topically per label recommendations, but at the minimum recommended dose of 0.12 mL/kg, delivering 1.44 mg/kg esafoxolaner, 0.48 mg/kg eprinomectin, and 10.0 mg/kg praziquantel. Cats assigned to the untreated control group were identically applied mineral oil at 0.12 mL/kg.

To detect the presence or absence of any treatment-related or unrelated health abnormality, health observations were conducted 1, 3, and 6 h after treatment application and daily afterwards, until the end of the study.

### Tick infestations

Each cat was infested in a random order, on Days −2, 7, 14, 21, 28, 35, and 42. To facilitate tick infestation, cats were sedated with an intramuscular injection of 0.1 mg/kg medetomidine and placed in an infestation chamber. Fifty (±4) unfed adult *A. maculatum* with a balanced sex ratio were placed on the lateral side of each sedated cat and care was taken to avoid the treatment application site. After a maximum of 4 h and full recovery from sedation (no reversion product was used), cats were returned to their housing enclosure.

### Tick counts

Tick counts and removals were performed 72 h following treatment and subsequent infestations (on Days 3, 10, 17, 24, 31, 38, and 45). For each count, ticks were identified visually or through fingertip palpation and hair parting of the whole body. After tick removal, the whole body was combed using a fine-toothed comb for a second check for tick presence (any tick found in a comb was classified as ‘attached’). Each tick was observed for signs of viability or mortality. A tick was considered alive when any level of motion or response to stimuli was detected. Ticks were classified as attached or free and live or dead. Protective clothing (e.g., gowns/coats, gloves, etc.) and combs were changed between each cat to prevent cross-contamination.

### Statistical analysis

The statistical unit was the individual cat. Differences in live attached tick counts between untreated control and treated cats were used to determine efficacy.

The efficacy at each time-point was calculated using the formula:

Efficacy (%)= 100 × (LSMc – LSMt)/LSMc,  where:

LSMc = least square mean number of live attached ticks in the untreated control group

LSMt = least square mean number of live attached ticks in the investigational veterinary product (IVP)-treated group

For each time-point, a linear mixed model was performed to test for a difference between the (untransformed) live attached tick counts in the IVP-treated and untreated control group. The model included treatment as a fixed effect and block as a random effect, at a 5% two-sided level.

The treatment was considered effective for the control of *A. maculatum*, when the following three criteria were met:

Adequate infestation, defined as ≥25% retention of live ticks in at least six control cats and at each time-point.
Calculated efficacy of ≥90%, at each time-point.
Statistically significant difference (*p* ˂ 0.05) between the tick numbers in the untreated control and treated group with a higher number of live ticks in the control group, at each time-point.

## Results

The efficacy results are detailed in Table 1.

**Table 1:** Efficacy of the investigational veterinary product (IVP) against *Amblyomma maculatum* infestations 72 h after treatment and subsequent weekly infestations.

Day	3	10	17	24	31	38	45
LS_C_ (AM untreated control group)	23.3	30.8	27.8	33.7	29.1	28.0	29.0
LS_T_ (AM IVP treated group)	0.3	0.2	0.3	0.1	1.8	1.1	3.3
% reduction^1^	98.7	99.4	98.9	99.7	93.8	96.0	88.6
*p*-value^2^	<0.0001	<0.0001	<0.0001	<0.0001	<0.0001	<0.0001	0.0013

1 The percent reduction was calculated as 100 × [(LS_C_ − LS_T_)/LS_C_], where LS_C_ was the least square arithmetic mean of the live attached tick counts among the untreated control cats and LS_T_ was the least square arithmetic mean of the live attached tick counts among the IVP treated cats.

2 *p*-value: Linear mixed model with treatment as a fixed effect and block as a random effect.

Inclusive of all time-points, the average number of live attached ticks found on the untreated control cats was 28.1 (i.e., 56.2% retention), and individually these numbers ranged from 13 (26%) to 43 (86%). This demonstrated a vigorous tick population and adequate infestations of cats throughout the study, with 100% of untreated cats adequately infested at each time-point.

The curative efficacy of a single application of NexGard^®^ Combo against existing tick infestation, 72 h after treatment was 98.7%. The preventive efficacy 72 h after weekly infestations over the following five weeks ranged from 93.8% to 99.4%.

Over the course of the study, tick bite reactions requiring, for some, topical treatment with an ointment combining antibiotics and corticosteroids were observed on several cats (8 cats from the untreated control group and 4 cats from the treated group), and the attending veterinarian recommended the removal of 7 cats (4 cats from the untreated control group and 3 cats from the treated group) mostly before the last infestation (on Day 42), for animal welfare considerations. No adverse reaction related to treatment was observed and no concurrent medication that may have interfered with the study results was used.

## Discussion

This is the first time that an induced infestation with *A. maculatum* is described in cats. The weekly reduction levels of live attached *A. maculatum* consistently exceeding 93% for five weeks in the treated cats provided a demonstration of immediate and sustained efficacy of one application of NexGard^®^ Combo against the infestations. This was supported by the high rate of live attached ticks in all untreated control animals 72 h after each infestation, demonstrating that the *A. maculatum* isolate used in this study was vigorous and well adapted to feline induced infestation. This high attachment rate also suggests that cats are competent hosts of *A. maculatum* in the field. It is noteworthy to observe that the adult *A. maculatum* bites in this study caused significant skin reactions to their feline host in several instances, as already described in other host species, for example in livestock [10, 23, 29]. Seven out of the 20 cats were removed before the end of the 5 weekly infestations for welfare consideration, demonstrating the direct pathogenic effect of this tick species. *Amblyomma maculatum* is a tick species infesting several mammalian species and of significant veterinary and human health importance, because it may cause significant inflammatory bite wounds and because it is a known vector of several pathogens [5, 11, 25, 32]. There is currently no feline product with a registered efficacy against *A. maculatum* in cats. The study described here supports the use of NexGard^®^ Combo to protect cats in areas endemic for *A. maculatum*.
